# A comparative study of tumour-on-chip models with patient-derived xenografts for predicting chemotherapy efficacy in colorectal cancer patients

**DOI:** 10.3389/fbioe.2022.952726

**Published:** 2022-08-16

**Authors:** Louis Jun Ye Ong, Shumei Chia, Stephen Qi Rong Wong, Xiaoqian Zhang, Huiwen Chua, Jia Min Loo, Wei Yong Chua, Clarinda Chua, Emile Tan, Hannes Hentze, Iain Beehuat Tan, Ramanuj DasGupta, Yi-Chin Toh

**Affiliations:** ^1^ School of Mechanical, Medical and Process Engineering, Queensland University of Technology (QUT), Brisbane, QL, Australia; ^2^ Centre for Biomedical Technologies, Queensland University of Technology (QUT), Brisbane, QL, Australia; ^3^ Department of Biomedical Engineering, National University of Singapore, Singapore, Singapore; ^4^ Institute for Health Innovation and Technology, National University of Singapore, Singapore, Singapore; ^5^ Laboratory of Precision Oncology and Cancer Evolution, Genome Institute of Singapore, A*STAR, Singapore, Singapore; ^6^ Biological Resource Centre, Agency for Science, Technology and Research (A*STAR), Singapore, Singapore; ^7^ Samuel Oschin Cancer Center, Cedars-Sinai Medical Center, Los Angeles, CA, United States; ^8^ National Cancer Centre Singapore, Singapore, Singapore; ^9^ Singapore General Hospital, Singapore, Singapore; ^10^ Experimental, Drug Development Centre, A*STAR, Singapore, Singapore; ^11^ Duke-NUS Graduate Medical School, Singapore, Singapore

**Keywords:** organ-on-chip (OoC), PDX (patient derived xenograft), dose response, 3D culture, microfluidic lab-on-a-chip, *in vitro*, *in vivo*

## Abstract

Inter-patient and intra-tumour heterogeneity (ITH) have prompted the need for a more personalised approach to cancer therapy. Although patient-derived xenograft (PDX) models can generate drug response specific to patients, they are not sustainable in terms of cost and time and have limited scalability. Tumour Organ-on-Chip (OoC) models are *in vitro* alternatives that can recapitulate some aspects of the 3D tumour microenvironment and can be scaled up for drug screening. While many tumour OoC systems have been developed to date, there have been limited validation studies to ascertain whether drug responses obtained from tumour OoCs are comparable to those predicted from patient-derived xenograft (PDX) models. In this study, we established a multiplexed tumour OoC device, that consists of an 8 × 4 array (32-plex) of culture chamber coupled to a concentration gradient generator. The device enabled perfusion culture of primary PDX-derived tumour spheroids to obtain dose-dependent response of 5 distinct standard-of-care (SOC) chemotherapeutic drugs for 3 colorectal cancer (CRC) patients. The *in vitro* efficacies of the chemotherapeutic drugs were rank-ordered for individual patients and compared to the *in vivo* efficacy obtained from matched PDX models. We show that quantitative correlation analysis between the drug efficacies predicted *via* the microfluidic perfusion culture is predictive of response in animal PDX models. This is a first study showing a comparative framework to quantitatively correlate the drug response predictions made by a microfluidic tumour organ-on-chip (OoC) model with that of PDX animal models.

## Introduction

One of the goals in precision oncology is to identify and administer effective treatment regimen suitable for individual cancer patients. Due to the nature of tumour heterogeneity arising from genetic (Chia et al., 2017; Lin et al., 2017; Wei et al., 2017) and non-genetic factors (Marusyk et al., 2012; Caiado et al., 2016; Sharma et al., 2018), cancer therapy is shifting from a “one-size-fits-all” treatment approach towards a tailored-approach in identifying effective treatment regimen for an individual patient. With a personalised approach in treating cancer, patients can benefit by undergoing effective treatment with minimal side effects. The use of patient-derived xenograft (PDX) models has been commonly reported in drug screening and to predict drug responses for individual patients (Votanopoulos et al., 2019; Antonia et al., 2020; Wu et al., 2021). Many PDX models retain primary histological and genetic signatures of their donor tumour and have demonstrated the ability to predict clinical outcomes. Hence, PDX models are being used for preclinical drug evaluation, biomarker identification, biologic studies, and personalised medicine strategies (Hidalgo et al., 2014). However, the use of PDX models is often time and cost intensive and is not scalable in terms of testing or validating multiple drugs and dosing. Given that the total cancer mortalities recorded almost 10 million cases worldwide in 2020, there remains an unmet need for a cost and time-efficient platform that could provide individualised early prediction for different combinations and dosages of existing anti-tumour drugs.

Microfluidic Organ-on-a-Chip (OoC) systems are widely regarded as a potential animal-alternative platform for drug screening applications. OoC systems leverage on microfabrication techniques to mimic different aspects of tissue microenvironment and physiology (shear stress and microenvironment) (Hattori et al., 2014; Short et al., 2017; Shuler, 2017) in a microfluidic device, which are modular and amenable to scale-up and multiplexing for drug testing applications. A plethora of tumour OoC devices have been developed to date with most utilising immortalised cancer cell lines (Du et al., 2018; Carvalho et al., 2019). The use of the immortalised cell lines fail to reveal the heterogeneity of individualized treatment response observed in the clinic (Katt et al., 2016). Additionally, with OoC systems in the early development stages, these devices either focus on reproducing the tumour microenvironment, such as the tumour vasculature (Tsai et al., 2017) and hypoxic core (Ayuso et al., 2019) or feature multiplexed designs with arrays of 3D tumour spheroids addressed by fluid manipulation networks (e.g., gradient generators, combinatorial mixers) to administer drugs at various concentrations or combinations simultaneously (Hung et al., 2005). The incorporation of primary patient tumour samples and/or PDX-derived tumour cells in microfluidic platforms could provide individualised drug testing but their degree of predictive capacity in comparison to PDX models remains unclear (Gheibi et al., 2017; Rodriguez et al., 2020). More importantly, there have not been systematic validation studies to ascertain whether short-term drug testing (< 1 week) using tumour OoC *in vitro* models are predictive of *in vivo* drug response, which takes a longer time (>4 weeks). This has in turn limited their routine application in cancer drug testing applications.

Here, we report the first instance of a direct *in vitro-in vivo* comparative study to develop a comparative framework for drug response predictions made from a tumour OoC models against that of matched PDX models in a patient-specific manner. PDX-derived 3D tumour spheroids were introduced into an Integrated Microfluidic Tumour Culture Array (IMITA) device, comprised of an 8 × 4 array of 3D tumour spheroid culture-chambers fed by a microfluidic concentration gradient generator. We demonstrate that the IMITA device can support perfusion culture of primary tumour spheroids isolated from PDX models generated from 3 colorectal cancer (CRC) patients, and simultaneously test the response of 5 standard-of-care (SOC) chemotherapies at 8 concentrations with 4 repeats. Importantly, the efficacy rank order of the 5 SOC drug combinations for individual patients predicted from the 3D spheroid cultures grown in the IMITA device correlated well with that obtained from the treatment of matched PDX models. Overall, this study demonstrates that microfluidic OoCs platforms, such as the IMITA device, offer a means to efficiently upscale drug testing with various drug combinations using minimal patient-derived or PDX tumour samples, and are predictive of *in vivo* response.

## Materials and methods

### Reagents

All chemicals and reagents were purchased from Sigma-Aldrich Pte Ltd., Singapore unless otherwise stated.

### Device design, fabrication, and assembly

The Integrated Microfluidic Tumour Array (IMITA) array was designed using AutoCAD (version 2017 Autodesk, United States). The IMITA device comprised a cell culture array with 32 circular chambers arranged as 8 × 4 (row × column). Each circular perfusion chamber was 2 mm in diameter and housed a cell culture chamber, which was 500 µm in diameter. The perfusion chambers were fed by an orthogonal network of microchannels, namely the cell seeding channels and the outputs of a concentration gradient generator. The volume of the cell culture chamber was defined by an array of micro-pillars (100 µm in length and 30 µm in width with an inter-pillar spacing of 20 µm), which was arranged into a circular cup. The circular micro-pillar array had a 250 µm wide opening, which faced the flow direction from the cell seeding inlet. Cells that were dynamically seeded into the device would be trapped by the micro-pillar array and packed at high density in the cell culture chamber. This physical trapping mechanism is a well-established method to form 3D spheroids in a non-cell type specific manner (Toh et al., 2007; Ong et al., 2017a; Ong et al., 2017b; Ong et al., 2019). The periphery of the perfusion chamber, orthogonal to the cell seeding inlet flow, was lined with pillars as cell filters at 100 µm in length and 30 µm in width with a 20 µm space. The 8 rows of culture chambers were connected to the outputs from a concentration gradient generator with 100 µm-wide serpentine channels. The device channel height was kept consistent at 100 µm.

The microfluidic devices were produced by moulding polydimethylsiloxane (PDMS) (Dow Corning, United States) on DRIE-etched silicon templates. The microfluidic device was capped with either a glass or PDMS substrate using plasma bonding (at 50 W, 20 sccm of O_2_, for 50 s) (FemtoScience, TN, United States) to a 75 cm × 50 cm glass slide.

The perfusion inlet and outlet of the device were connected *via* Tygon^®^ tubing (ND 100-80, Saint-Gobain, United States) to 3 ml fluid dispensing reservoirs (Nordson EFD, United States) and 1-way stopcocks with Luer connections (Cole-Parmer, United States). Quick connect Luer adaptors (Upchurch Scientific, United States) connected the tubing to the stopcocks. Prior to assembly, all parts were sterilized by autoclaving at 121°C for 20 min except for the stopcocks, which were sterilized with 70% ethanol for 1 h before rinsing with sterile 1X PBS. The assembled device was then mounted on a 3D printed support frame, printed with an Objet260 Connex3 Printer (Stratasys, United States), and using acrylonitrile butadiene styrene (ABS) plastic, whereby the inlet and outlet reservoirs were supported on a 3D printed insert with 2 support pillars with a height difference of 6.3 cm.

### Computational fluid dynamics simulation

CFD simulation was performed using Autodesk CFD (Autodesk, United States). The device geometry was adapted from previously established design (Ong et al., 2017b). Different mesh sizes were used in the simulations until the difference between two successive mesh scales was less than 5% (following previously established framework). The medium viscosity was assumed to be the same as that of water (0.001003 Pa·s). Flow velocity and wall shear stress at the cell culture chambers were determined at hydrostatic pressure generated when the height difference between the inlets and outlet media reservoirs were set at 6.3 cm. For mass transport studies, simulations were carried out at steady state. Steady states were determined by running the simulations with across time until the simulated mass fractions at each culture chambers were less than 5% difference. The boundary conditions were set by with the outlet pressure at 1 atm and inlet flow rate of 0.02 ml·h^−1^.

### Generation and passaging of patient-derived colorectal cancer patient derived xenograft

Tumour samples were obtained from patients’ post-surgery with informed patient consent in accordance with SingHealth Centralised Institutional Review Board (CIRB: 2015/2165). Tumours were minced into ∼1 mm^3^ fragments and suspended in a mixture of 5% Matrigel (Corning, United States) in DMEM/F12 (Thermo Fisher, United States). The tumour fragment mixtures were then implanted subcutaneously into the left flanks of 5–7 weeks old female NSG (NOD-scid IL2Rgammanull, NOD-scid IL2Rgnull, NOD scid gamma) (NOD.Cg-Prkdcscid Il2rgtm1Wjl/SzJ, Jackson Laboratory, stock no. 005557) mice, using 18-gauge needles. Tumours were excised and passaged when they reached 1.5 cm^3^ (calliper measurements done at 3 times a week). For passaging, tissues were cut into small fragments of 1 mm^3^ prior to resuspension in a 20% Matrigel/DMEM/F12 mix, before subcutaneous inoculation of tumour fragments into 5–7 weeks old NSG mice. Animals were housed in individually ventilated cages in the Biological Resource Centre, A*STAR, Department 2. Room lighting was set to a 12-h light-dark cycle as recommended by the National Advisory Committee for Laboratory Animal Research (NACLAR). Animals were provided with an irradiated Altromin 1324 diet and autoclaved water, *ad libitum*. Protocols for all the animal experiments described were approved by the A*STAR Biological Resource Centre (BRC) Institutional Animal Care and Use Committee (IACUC) under protocol #171207.

### Drug preparation and *in vivo* drug treatment

5-Fluorouracil (5-FU, CAS#: 51-21-8, AK Scientific) and Irinotecan (CPT-11, CAS#: 136572-09-3, Active Biochem) were prepared to a concentration of 5 mg·ml^−1^ in 50% Polyethylene Glycol 400 (Sigma) and administered at a dosage of 50 mg·kg^−1^ i. p. Once a week (IRI) and twice a week (5-FU). Oxaliplatin (OXA, CAS#: 61825-94-3, Active Biochem) was prepared to a concentration of 1 mg·ml^−1^ with the same vehicle and administered at a dosage of 5 mg·kg^−1^ i. p. Once a week. Fluorouracil + Oxaliplatin (5-FU + OXA) and Fluorouracil + irinotecan (5-FU + CPT-11) were prepared with same concentration and dosed with the schedule of their single agent counterpart with 5-FU being administered first. Control groups for all compounds were treated in their corresponding vehicle (50% Polyethylene Glycol 400), following standard procedures (Pandey et al., 2017), in the absence of compounds. All treatments were given for 6 consecutive weeks.

The length and width of tumours were measured by calliper once every 2 days. Tumour volumes were determined using the following modified ellipsoidal formula: Tumour volume = ½ (length × width^2^). Mice were euthanised when tumours in the control group reached 2.0 cm^3^. The tumour growth inhibition percentage (TGI%), the ratio of the change in treated tumour volume (ΔT) to the average change in control tumour volume (ΔT/Average ΔC) at each time point, was used to determine drug treatment efficacies, and calculated as follows:

$$\text{Tumour}\;\text{volume}\;\text{of}\;\text{treatment}\;\text{group,}\;\text{T}\;=\;\text{½}\;\left(\text{length}\;\text{×}\;{\text{width}}^{2}\right)$$ (1)

ΔT = Tumour volume of drug-treated group on study day−Tumour volume on initial day of dosing.

C = Tumour volume of control group.

ΔC = Tumour volume of control group on study day−Tumour volume on initial day of dosing.

Average ΔC = Average change in tumour volume across the control-treated group

TGI % = ΔT/Average ΔC X 100 (2)

### Isolation and seeding of patient derived xenograft-derived tumour cells into integrated microfluidic tumour array device

Tumour tissues excised from PDX were minced prior to enzymatic dissociation using 4 mg ml^−1^ collagenase type IV (Thermo Fisher, United States) in DMEM/F12, at 37 °C for 2 h. Cells were washed using the cyclical treatment of pelleting and resuspension in phosphate-buffered saline (Thermo Fisher, United States) for 3 cycles. The final cell suspensions were strained through 70 µm cell strainers (Falcon, United States), prior to pelleting and resuspension in RPMI (Thermo Fisher, United States), supplemented with 10% foetal bovine serum (Biowest, United States), and 1% penicillin-streptomycin (Thermo Fisher, United States). Cells were kept in a humidified atmosphere of 5% CO_2_ at 37°C. Cells were routinely screened for mycoplasma contamination using the Venor^®^GEM OneStep mycoplasma detection kit (Minerva Biolabs, United States).

### Seeding and culture of patient derived xenograft-derived colorectal cancer cells in integrated microfluidic tumour array device

The culture medium was formulated using RPMI 1640 Medium (Gibco, United States) supplemented with 10% foetal bovine serum (Biowest, United States), and 1% Pen-Strep (ThermoFisher Scientific, United States). Cell seeding was initiated by withdrawing a cell suspension (cell density fixed at 10 million cells mL^−1^) from the cell reservoir through the common outlet with a withdrawal syringe pump at a flow rate of 0.03–0.08 ml h^−1^ (KD Scientific, United States), and the perfusion inlets and outlets closed. Once the microfluidic device was almost full, the cell-free media was added into the cell seeding inlet to wash off cells not trapped within the central chamber before closing the seeding inlet and outlet. The perfusion outlet reservoir was connected to the stopcock at the common outlet and medium perfusion was initiated by turning on the perfusion inlet and the common outlet of the device. The seeded device was then mounted on the 3D printed support and incubated at 37 °C with 5% CO_2_ for 24 h with the culture medium before any drug dose-response study.

### Drug dose-response study of patient derived xenograft-derived colorectal cancer in integrated microfluidic tumour culture array device

Post 24 h of perfusion culture with culture medium, the medium in all perfusion inlets and outlets were removed by pipetting. 5 different drug combinations were tested for this study: Fluorouracil (5-FU), Oxaliplatin (OXA), irinotecan (IRI), Fluorouracil + Oxaliplatin (5-FU + OXA), and Fluorouracil + irinotecan (5-FU + IRI). All compounds were dissolved in DMSO (Sigma-Aldrich, United States). For each drug dose-response, 500 μM of the drugs were diluted using DMSO into the cell culture media and loaded into the drug inlet medium reservoir. For combinatorial drugs, 500 μM of the individual drugs were mixed into the cell culture media. The cell culture medium infused with 1% (v/v) DMSO was loaded into the diluent medium reservoir. The seeded devices were then incubated for 3 days at 37 C with 5% CO_2_ before cell viability staining.

### Cell viability staining

*In situ* labelling of viable cells was performed using Calcein AM (ThermoFisher Scientific, United States). Culture medium containing 2 µM Calcein AM was perfused at 0.08 ml·h^−1^ from both perfusion inlets using a syringe pump for 1 h at 37°C and 5% CO_2_. The devices were then washed with the culture medium at 0.08 ml·h^−1^ for 30 min before viewing with the fluorescence microscope (Nikon, Japan) anda CoolLED pE-2 LED-excitation light source (CoolLED, United Kingdom) at wavelength of 470 nm for Calcein AM. For each chamber, the transmission image and fluorescence image were taken for each chamber. Exposure time was kept at 300 ms for all fluorescent microscopy imaging steps.

### Image quantification

All microscope images were processed as 16-bit images for consistency. Transmission images of the CRC organoids were used to identify and measure the area of the organoid within the chambers. Cells located outside the cell culture chambers were not considered. The identified organoid area was used to create a mask in ImageJ (NIH, United States) and overlaid against the fluorescence images. The mean fluorescent intensities of the mask-overlaid fluorescent images were quantified, and then normalised to the measured organoid area. Across the different concentration of drugs down the row, the area-normalised fluorescence intensities for each chamber where normalised again to the mean normalised intensity measured from the chambers in the first row which served as a control Eq. 1.

$$\mathrm{Cell}\;\mathrm{viability}\;\%\;=\;\frac{\left({\frac{\mathrm{Intensity}}{\mathrm{Spheroid}\;\mathrm{area}}|}_{\mathrm{treated}\;\mathrm{chamber}}\;\right)}{\left(\frac{\sum {\frac{\mathrm{Intensity}}{\mathrm{Spheroid}\;\mathrm{area}}|}_{\mathrm{non}-\mathrm{treated}\;\mathrm{chamber}\;\left(\mathrm{row}\;1\right)}}{4}\right)}\times 100\%$$ (3)

### Statistical analysis

All results were obtained from at least 3 independent experiments with values expressed as mean ± standard deviation (SD). Curve fitting was undertaken with a GraphPad PRISM 8.0 (GraphPad, United States) using a 4-parameter variable slope, inhibition dose-response fitting to identify the IC_50_ values. A two-tailed Pearson correlation was computed using the GraphPad PRISM 8.0. All graphs were plotted on GraphPad PRISM 8.0.

## Results and discussion

### Design of the integrated microfluidic tumour array device

**FIGURE 1:**
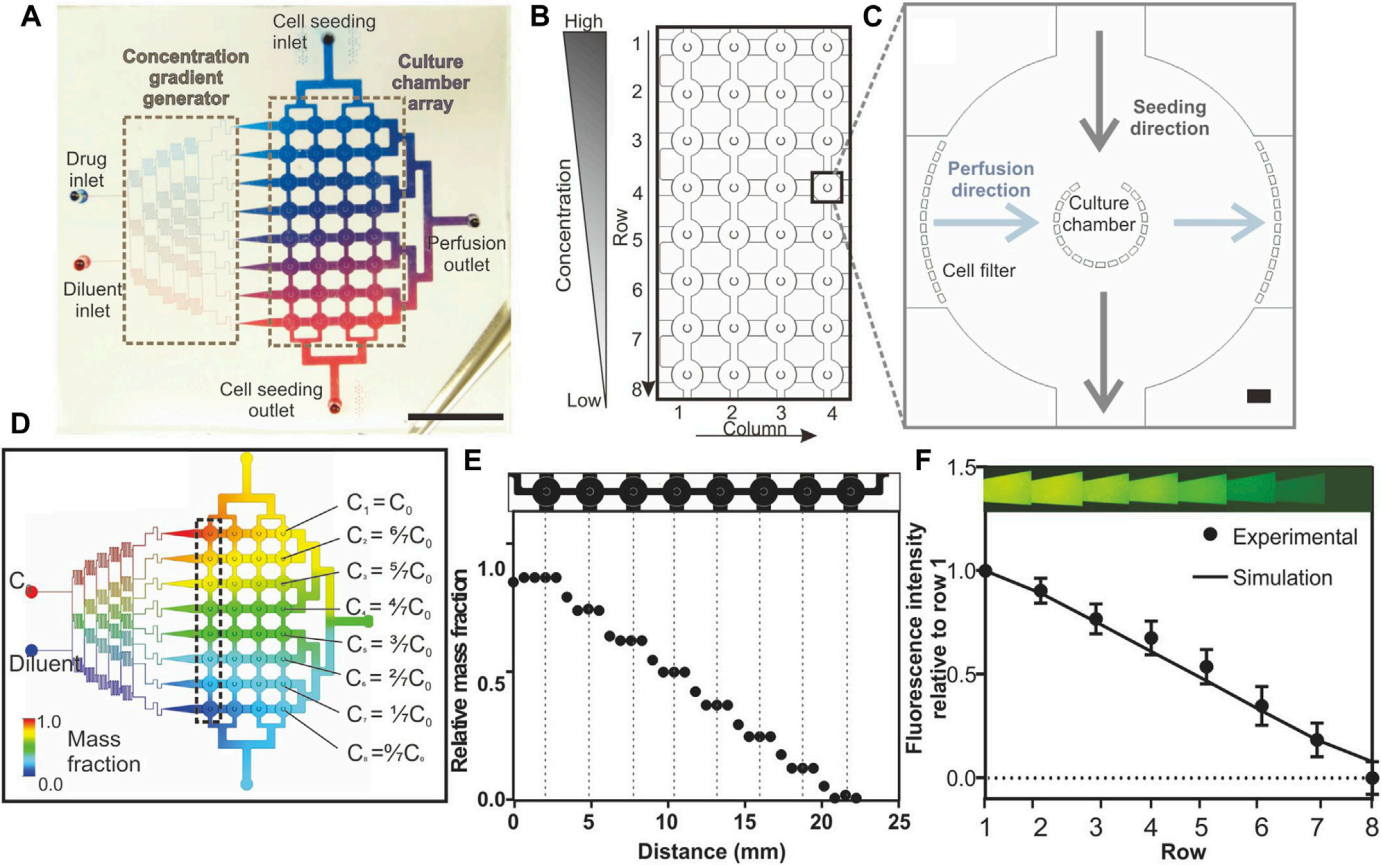
Design and characterisation of the Integrated Microfluidic Tumour Array (IMITA) device for multiplexed 3D cell cultures. **(A)** The IMITA device consisted of two functional blocks (Lin et al., 2017): concentration gradient generator; and (Wei et al., 2017) cell culture array connected by two orthogonal flow circuits for cell seeding and medium perfusion. Scale bar = 1 cm. **(B)** The cell culture array consisted of 32 cell culture chambers arranged in 8 rows by 4 columns. **(C)** Each cell culture chamber comprised a cup-shaped micropillar array with 20 µm gaps where the opening faced the seeding flow circuit to trap the incoming cells. A series of micropillars act as cell filters along the medium perfusion direction to prevent cell clogging during cell seeding. Scale bar = 100 µm. **(D)** CFD simulation showing 8 mass concentrations generated by a linear concentration generator, which were fed into each row of the IMITA device at steady state when operating at 0.02 ml h^−1^. **(E)** Simulated relative mass fractions as a function of distance along a single row of cell culture chamber [box indicated in 1(d)] at steady state operating condition. Mass fraction within a single cell culture chamber remained constant while a linear decrease was observed across each chamber in a row. **(F)** Experimental validation of the concentration gradient generator using rhodamine fluorescent probe. Data are averages of 3 experiment measurements ± standard deviations.

The IMITA device serves as a proof-of-concept multiplexed OoC platform, that can provide a suitable 3D tumour microenvironment to support the growth of primary PDX-derived tumour cells while being amenable to conducting dose response studies of chemotherapy drugs. The device was designed to handle parallel tumour spheroid cultures in an 8 × 4 array of culture chambers so that 32 tumour samples could be maintained simultaneously (Figure 1A). The culture chambers were individually addressed by 2 orthogonal flow networks similar to the design reported by Hung et al. (2005) so as to facilitate operation switching from a cell seeding mode to a perfusion culture mode (Figures 1A,B).

The 8 × 4 cell culture array (Figure 1B) served as the intersection point for the cell seeding flow circuit and perfusion culture circuit. Each cell culture chamber consisted of a micro-pillar array, surrounded by fluid perfusion (Figure 1C), and adapted from a prior design (Ong et al., 2017b; Ong et al., 2017a). This design allowed cells introduced from the cell seeding inlet to be physically packed and remodelled into 3D tumour spheroids within the chamber. Additional pillar arrays were incorporated at the inlets of the medium perfusion networks, surrounding the cell culture chamber peripheries, to prevent clogging of cells along the perfusion flow circuit and to ensure uniform flow during perfusion culture. The radius of the culture chambers were set at 250 μm to ensure that the mass transport distance of oxygen and nutrients can support long term perfusion cultures as previously demonstrated (Ong et al., 2017a; Ong et al., 2017b).

The perfusion circuit consisted of 2 inlets (1 drug and 1 diluent inlet) connected to a concentration gradient generator (Figure 1A). Branching channels were added following the established design guide reported by Dertinger et al. (2001) in order to generate a linear gradient across the 8 concentration outputs (Figure 1D). Since the device relied on constant laminar flow to maintain the concentration gradient in the cell culture array, we performed CFD simulation to determine whether the desired concentration gradient can be maintained across the entire cell culture array when operating at a designated perfusion flow rate (Figure 1D). Different mesh scale sizes were investigated (Supplementary Figure S6A) with less 3% difference in simulated flow rates (Supplementary Figure S6B). For the subsequent work, a medium scale was used to save computing time. We observed that there was a linear decrease in steady state mass fraction (concentration) across every row of cell culture chambers, although the concentration within each chamber could be Figure 1E kept constant (Figure 1E). We then experimentally verified the concentration gradient of a fluorescent probe across a row of cell culture chambers in the IMITA device (Figure 1F). The concentration gradient across each row showed a linear drop in concentration by a factor of 0.1429 with C_n_ = (1-(n-1)/7) x C_0_ (with n starting from 1 to 8, representing the output rows); across each row of output (*R* ^*2*^ = 0.9863) and concurred with our simulation results. By changing the inlet drug concentration, C_0_, we were able to modify the range of drug concentrations. Our subsequent drug dose response study examined the ML drug dose response with 8 drug concentrations with C_0_ = 500 μM (C_1–8_ = 500, 378.60, 375.10, 285.70, 214.30, 142.85, 71.40, and 0.00 μM respectively) for all drugs.

### Seeding and perfusion culture of patient derived xenograft-derived spheroids in the integrated microfluidic tumour array device

The operation of the IMITA device was designed to be minimally dependent on external pumps and compatible with a standard biological lab using an established pump-free perfusion culture setup (Supplementary Figure S1) to drive fluid flow (Ong et al., 2017a). Using CFD simulation, we estimated a pressure head of 63 mm H_2_O was sufficient to drive a perfusion rate of 0.02 ml·h^−1^ (Supplementary Figure S2) which corresponded to a low wall shear stress of 0.04 dyne·cm^−2^ at the cell culture chamber (Supplementary Figure S3, *). While this was a relatively low flow rate, we verified that the Péclet number (*Pe*) of model chemotherapeutic drugs in the cell chambers were in the convection dominated transport regime (Pe >> 1) and at least one order of magnitude higher than that in the seeding channels linking adjacent cell chambers in a row (Supplementary Figure S3A,B). This indicated the tumour spheroids in each cell culture chamber would be exposed to drug concentrations in accordance with the output of the concentration gradient generator (Figures 1E,F) with minimal diffusive mixing between adjacent chambers in the same row *via* the cell seeding channels.

**FIGURE 2:**
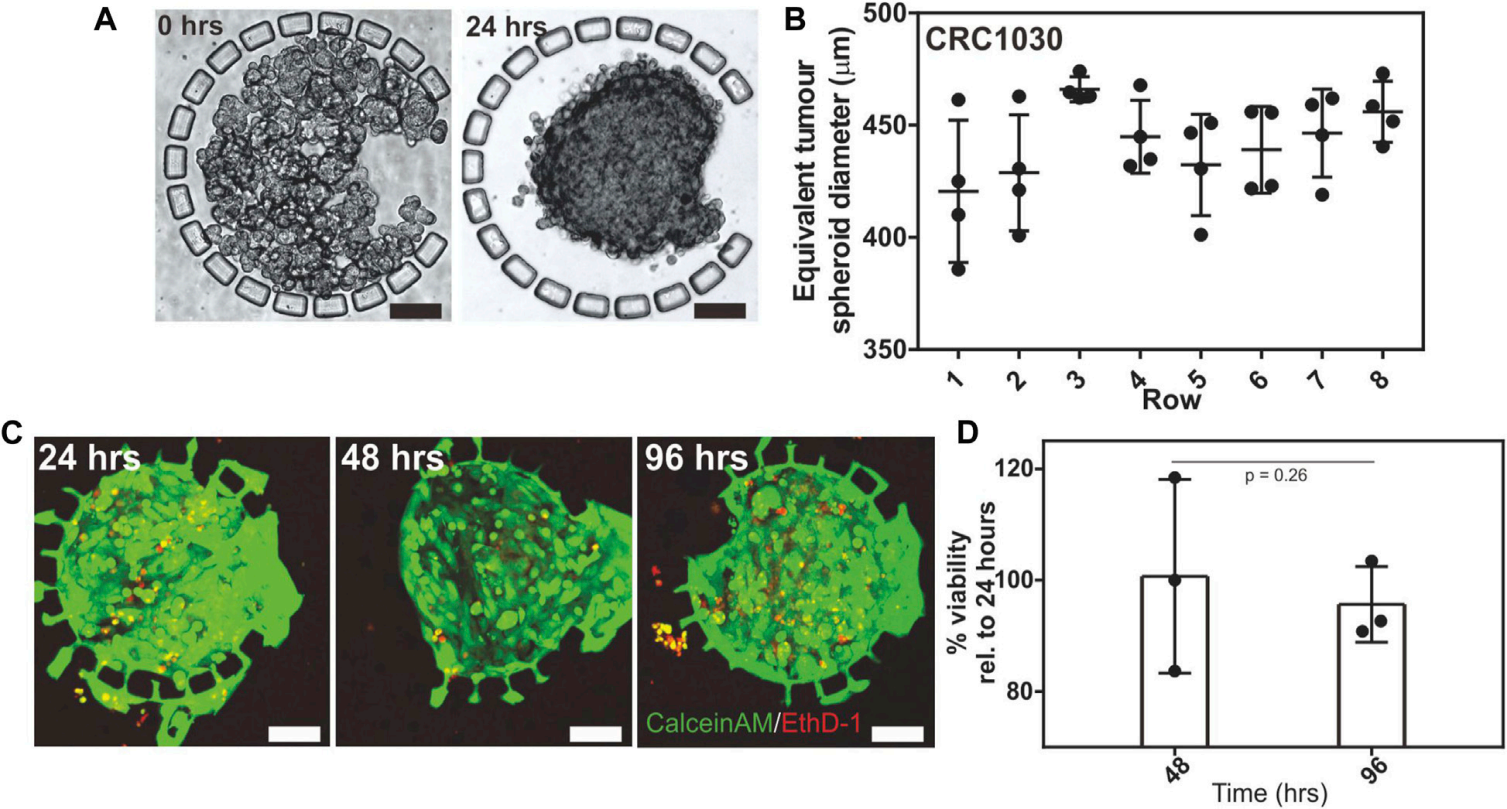
Seeding and perfusion culture of PDX derived tumour spheroids within the IMITA device. Transmission images showing: **(A)** CRC1030 PDX derived tumour cells seeded and trapped by the micropillar array in the cell culture chamber; **(B)** seeded cells remodelled into a 3D tumour spheroid after 24 h of perfusion culture. Scale bars = 100 µm; **(C)** Quantification of PDX derived tumour spheroid size distribution across all rows of the IMITA device after 24 h of perfusion culture (One-way ANOVA, *p* = 0.0891). The spheroid’s equivalent diameter was estimated from the spheroid’s area; **(D)** Live (CalceinAM, green)-dead (EthD-1, red) staining of the seeded PDX derived tumour cells within the IMITA device after 96 h of perfusion culture. Scale bar = 100 µm.

To investigate the seeding and consistency in which 3D tumour spheroids can be formed in the IMITA device, we seeded isolated PDX tumour cells (patient CRC1030) into the array followed by 4 days of perfusion culture. We observed that the PDX tumour cells underwent remodelling, changing from a loosely packed configuration (Figure 2A) to a densely packed structure (Figure 2A) within 24 h of culture. This observation was in agreement with our previous results on micropillar-mediated cell aggregation (Ong et al., 2017a; Ong et al., 2017b), and demonstrated that the IMITA device was able to create a 32 plex array of PDX-derived 3D packed tumour cells. Subsequent drug testing studies were therefore initiated 24 h post-seeding to allow for the formation of compact 3D tumour cells in the IMITA device.

We also quantified the size distribution of the PDX-derived tumour spheroids in the IMITA device after 48 h of perfusion culture. We observed that the size of the tumour spheroids remained relatively uniform after 48 h of culture across the array, with an average diameter of 441.71 ± 14.8 µm (Figure 2B). A comparison of the tumour spheroid diameter across each row of the IMITA array identified spheroid sizes to be relatively similar (One-way ANOVA, *p* = 0.0891), suggesting that cells seeded into the device can be uniformly distributed across all channels in the cell seeding flow circuit. The relative uniformity in the spheroid size is ideal for drug dose response studies as it ensures consistent diffusion lengths as the drugs diffused into the tumour spheroid. This uniformity will also aid in future automated measurement and analysis such as viability imaging and quantification, which facilitates multiplexed experiments. We further characterised the viability of the PDX-derived tumour spheroids within the IMITA device to investigate the duration over which the device can maintain the (Ong et al., 2020) spheroids. It was observed that the spheroids can remain viable for up to 96 h (4 days) of culture (Figure 2C). Cell viabilities that were normalised to that of Day 1 was 100.7 ± 8.6% and 95.6 ± 5.6% at Day 2 and Day 4, respectively (Figure 2D). This observation indicated the IMITA device could support 24 h of 3D tumour tissue remodelling followed by 3 days of drug treatment, which corresponded well to the time frame (< 1 week) that are typical of acute *in vitro* drug testing (Oleaga et al., 2016; Gheibi et al., 2017; Pandya et al., 2017; Virumbrales-Muñoz et al., 2019).

### Responses to standard-of-care chemotherapies for colorectal cancer patients

**FIGURE 3:**
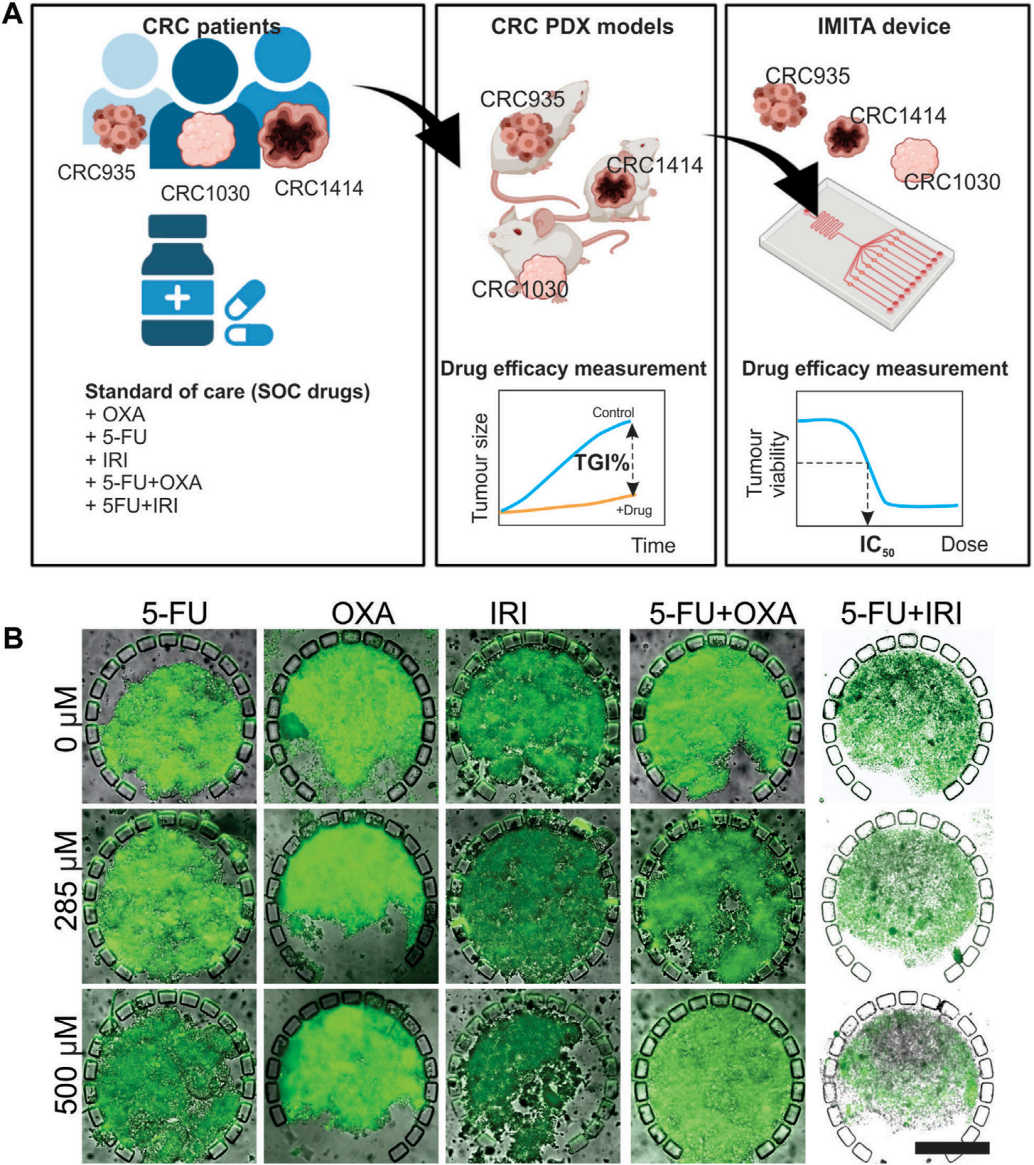
*In vivo-in vitro* comparative study to evaluate the efficacy of 5 standard-of-care (SOC) chemotherapies for 3 CRC patients. **(A)** Experimental design. The 5 SOC chemotherapies tested were 5-Fluorouracil (5-FU), Oxaliplatin (OXA), irinotecan (IRI), 5-Fluorouracil + Oxaliplatin (5-FU + OXA or 5-FU/OXA), and 5-Fluorouracil + irinotecan (5-FU/IRI). Figure created with BioRender.com. **(B)** Calcein-AM fluorescent images overlaid with transmission images showing viability of CRC 1414 tumour spheroids in IMITA devices after 24 h of treatment with 5 SOC chemotherapies at selected concentrations. Magnification = ×10. Scale bar = 250 µm.

Next, we performed individualised drug screening using patient-specific PDX-derived spheroids, derived from 3 colorectal cancer (CRC) patients (CRC935, CRC1414 and CRC 1030), maintained in the IMITA device. A parallel *in vivo* drug response study conducted with PDX models from the same patients is important to facilitate the quantitative drug response comparison between the two models (Figure 3A). Specifically, it can inform whether the purported advantage of faster screening time using *in vitro* tumour OoC models could compromise the prediction accuracy of *in vivo* testing, which typically spans more than 4 weeks (Ahmed et al., 2019). 5 standard-of-care (SOC) chemotherapies for CRC were evaluated, namely Fluorouracil (5-FU), Oxaliplatin (OXA), irinotecan (IRI), Fluorouracil + Oxaliplatin (5-FU + OXA, 5-FU/OXA), and Fluorouracil + irinotecan (5-FU + IRI, 5-FU/IRI). Two standard quantitative readouts, namely half-maximal inhibitory concentration (IC_50_) and tumour growth inhibition percentage (TGI%), were used to indicate the *in vitro* and *in vivo* anti-tumour efficacies, respectively, following our previous work (Ahmed et al., 2019).

For the IMITA device, tumour cells were isolated from PDXs, seeded into the device, and perfusion cultured for 24 h before initiating drug treatment for 3 days at 8 concentrations ranging from 0 to 500 μM. This range of drug concentration was chosen to include the maximum serum concentration (C_max_) values of the 5 SOC drugs in human, which range from 4.96 µM for (OXA) to 426 µM (5-FU) (Liston and Davis, 2017). The drug exposure period was chosen to represent the mean time period of drug screening models in microfluidic tumour cultures (Oleaga et al., 2016; Gheibi et al., 2017; Pandya et al., 2017; Virumbrales-Muñoz et al., 2019). The assay readout to assess drug responsiveness was selected to balance between accuracy of measuring anti-tumour effects and amenability to high throughput drug screening operations. To this end, we imaged and quantified cell viability of each spheroid using 2D wide-field fluorescence imaging as readouts to indicate for drug responses. Measurement of drug-induced changes in tumour cell viability is widely employed as a readout in drug screening studies (Green and Leeuwenburgh, 2002; Kim et al., 2012; Dereli-Korkut et al., 2014). Moreover, by measuring tumour cell viability instead of changes in tumour spheroid size, we can overcame a problem encountered by previous studies on microfluidic tumour spheroid cultures, whereby a portion of dead cells can remain lodged within the spheroids, and in turn result in false negative results (Gheibi et al., 2017) (Figure 3B). Quantification of spheroid viability was performed using 2D projection fluorescent intensity images to reduce the image acquisition and processing time per sample. We have previously verified that quantification results from 2D projection images of cell viability staining in 3D tumour spheroids concurred well with 3D volumetric quantification results obtained from 3D confocal image stacks (Ong et al., 2020). Using this assay readout, we could obtain response data from over 600 test samples (i.e., 5 drugs × 32 chambers per device × 4-5 devices per drug) within a relatively short period of time to generate the dose response curves of each SOC drug. This allows for the estimation of IC_50_ values, which were used as the primary parameter to indicate anti-tumour efficacies of the SOC drugs, where a lower IC_50_ indicates for higher anti-tumour efficacy.

**FIGURE 4:**
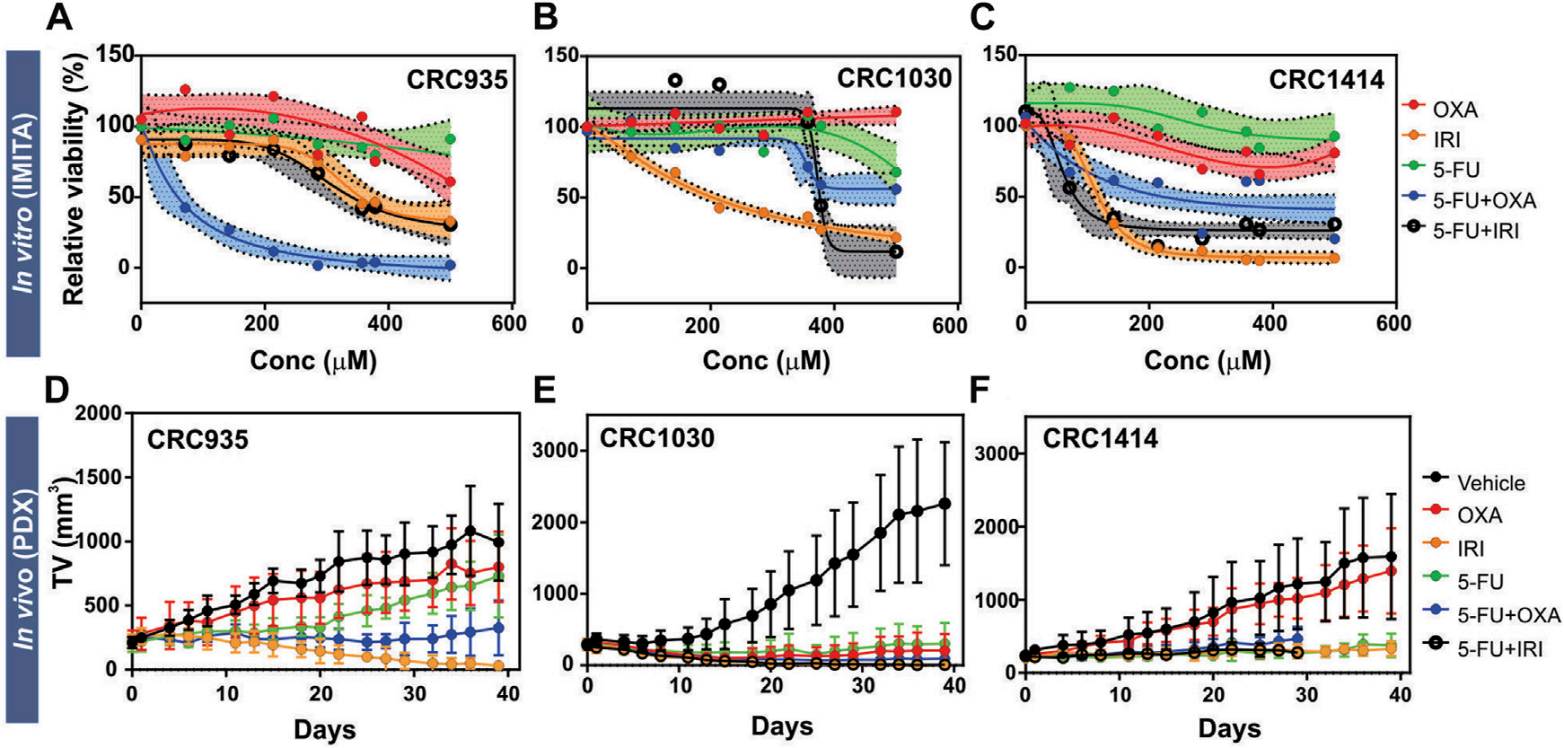
Treatment responses of 5 SOC chemotherapies for 3 CRC patients (CRC935, CRC1030, and CRC1414) obtained from *in vitro* (IMITA) and *in vivo* (PDX) models. **(A–C)** Dose-response curves obtained after 3 days of drug treatment in the IMITA devices. Fitted dose-response curve obtained using *n* > 3 devices with shaded area representing 95% confidence interval. Two-way ANOVA analysis yielded *p* = 0.002 for CRC935, *p* < 0.0001 for CRC1030 and CRC1414. **(D–F)** Tumour growth rates in PDX models. 5-Fluorouracil and IRI were administered at a dosage of 50 mg kg^−1^. OXA was administered at a dosage of 5 mg kg^−1^. 5-FU + OXA and 5-FU + IRI were prepared with same concentrations. Data are averages of *n* > 3 animals with ± standard deviation.

We observed differential responses for SOC chemotherapies across the IMITA devices housing PDX tumours derived from different CRC patients (Figures 4A–C). For all cases, the PDX tumour spheroids from CRC935, CRC1030 and CRC1414 did not respond to single Oxaliplatin (OXA) and 5-fluorouracil (5-FU) treatments but had improved sensitivities when both drugs were combined (5-FU + OXA). These observations are in agreement with previous clinical studies reporting better objective response and patient survival rates with combinatory drug treatments (Goldberg et al., 2007). Notably, the extent of sensitivity towards 5-FU + OXA treatment was variable between the 3 patients. Patients CRC935 and CRC1414 showed more than 50% reduction in tumour viability at drug concentrations above 68.53 μM and 225 μM, respectively. However, CRC1030 was more resistant and still had ∼50% tumour viability when treated at the highest (i.e., 500 μM) drug concentration (Figure 4B). Irinotecan (CPT11) was more effective as a single-drug treatment compared to OXA or 5-FU. Patients CRC1030 and CRC1414 were more sensitive to the CPT11 treatment, where tumour viability dropped to below 50% at drug concentrations as low as 207 μM and 114 μM, respectively, whereas CRC935 required a slightly higher concentration of 326.3 μM to reduce tumour viability to below 50% (Figure 4A). Notably, combination of CPT11+5-FU (5-FU/IRI) did not confer additional anti-tumour efficacy compared to the CPT11 treatment alone, except for CRC1414 (Figure 4C). Overall, we observed variability in the drug responses measured from the IMITA devices for different patients. This variability indicates the IMITA devices could maintain the patient-specific tumour characteristics of PDX cells, and therefore their drug responses over a period of 4 days.

**FIGURE 5:**
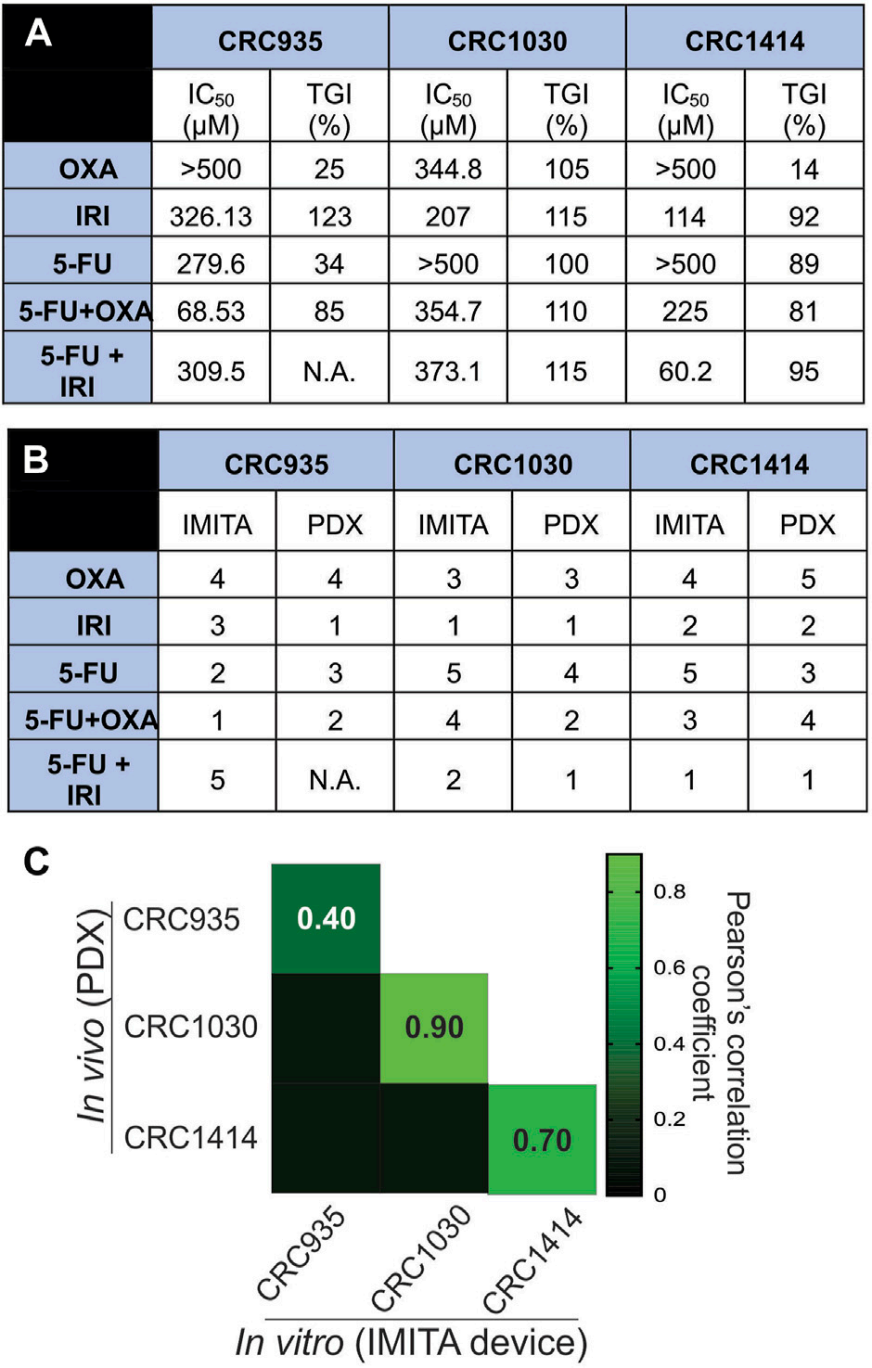
*In vitro-in vivo* drug response correlation. **(A)** TGI values from *in vivo* PDX models and IC_50_ values of dose response curves obtained from *in vitro* IMITA devices for 3 CRC patients. **(B)** Patient-specific drug efficiency ranking using *in vivo* PDX models and estimated IC_50_ values using the IMITA device. Rank 1 denotes most effective drugs (lower IC_50_ values for IMITA and higher TGI% for PDX). **(C)** Pearson correlation matrix comparing drug efficacy ranking estimated from the *in vivo* PDX models to the IC_50_ values from the *in vitro* IMITA devices.

The inter-patient variability of inherent tumour characteristics across the 3 CRC patients was evident from the PDX models. Of the control animals, CRC1030 exhibited the fastest tumour growth rate (Figure 4E) whereas CRC935 had the slowest (Figure 4D). Notably, CRC1030 PDX was the most susceptible to chemotherapy (Figure 4E), where the TGIs for all 5 SOC therapies were >100% (Figure 5A). CRC935 and CRC1414 PDXs both exhibited some resistance to specific therapies, especially for OXA treatment alone (TGI for CRC935 = 25%; TGI for CRC1414 = 14%) (Figures 4D,F, 5A). CRC935 PDX only responded to CPT11 and 5-FU + OXA treatments (Figure 4D) while CRC1414 was susceptible to CPT11, 5-FU and 5-FU + CPT11 (Figure 4F). The variation in drug responses across different patients is consistent with a plethora of clinical studies showing inter-patient heterogeneity in cancer response, which further strengthens the case for a personalised treatment approach to cancer therapy.

### *In vitro-in vivo* correlation of colorectal cancer chemotherapy efficacy prediction

The quantitative correlation between *in vitro* drug response observed in the IMITA device over a 4-day period and that of the PDX models over a 40-day period provides insightful window of opportunity to identify the degree of similarity between *in vitro* tumour OoC models and *in vivo* PDX models. Since the conventional measurement metrics to indicate treatment efficacies are different for *in vitro* (i.e., IC_50_ extracted from dose response curves at fixed timepoint) and *in vivo* models (i.e., TGI extracted from tumour growth rate at fixed dose), we first ranked the SOC therapies in terms of anti-tumour efficacies before performing correlation analysis. Using IC_50_ values obtained from the IMITA dose-response studies (Figure 5A), we ranked the 5 SOC therapies from most (rank 1) to least effective (rank 5), where a lower IC_50_ value would indicate a more effective treatment (Figure 5B). Similarly, the ranking of PDX animal models was based on their respective TGIs (Figure 5A) with most effective drugs resulting in a higher TGI (rank 1) and least effective (rank 5) drug with a lowest TGI value (Figure 5B). Subsequently, we determined the Pearson’s correlation coefficient between the efficacy rankings obtained from the IMITA *in vitro* model and the *in vivo* PDX models to assess how well they correlated using in-built tools in GraphPad PRISM 8.0. The drug efficacies ranking from *in vitro* IMITA array showed good agreement with that ranked using *in vivo* PDX models for CRC1030 and CRC1414 with a correlation coefficient of 0.90 and 0.70, respectively (Figure 5C). However, the ranking prediction for CRC935 showed only a correlation coefficient of 0.40 compared with the *in vivo* PDX model (Figure 5C) due to the fact that CRC935 was only susceptible to 5-FU + OXA (5-FU/OXA) treatment in the IMITA device whereas the IRI-based treatments were also effective in CRC PDXs models.

Despite limitations associated with cost, time and engraftment rates (Murayama and Gotoh, 2019), PDX models are still considered the “gold standard” to predict patient responses to a particular SOC treatment regime (Pompili et al., 2016). Therefore, while tumour OoCs have the potential to achieve personalised chemotherapeutic testing within a short period of time (i.e., ∼days versus months in animal testing), it is still critical to benchmark their drug response predictions to that of PDX models. Notably, our study results demonstrated that the tumour OoC platform, as exemplified by the IMITA device, was indeed able to capture patient-specific variability in drug responses (Figure 4A), just like PDX models (Figure 4B). More importantly, we extended prior research that incorporated primary or PDX tumour cells into OoC systems to account for patient-specificity (Pan et al., 2015; Gheibi et al., 2017) by quantitatively comparing the drug response predictions made by the *in vitro* IMITA device and *in vivo* PDX models. By incorporating PDX tumour cells obtained from 3 CRC patients, we showed that in 2 of the 3 patients, the IMITA devices could closely predict the rank-order of 5 SOC chemotherapies with high concordance to their PDX counterparts (Figure 5C). While the patient sample size is relatively small, this proof-of-concept study has established a quantitative framework to perform correlation analysis between *in vitro* and *in vivo* models even though they utilise different measuring metrics (e.g., IC_50_ and TGI) to indicate anti-tumour efficacies.

For *in vitro* models to be a suitable alternative in drug screening platform, it is necessary for the established model to be able to recapitulate physiological microenvironment conditions, such as 3D architectures (Short et al., 2017), fluid induced-shear stress (Mitchell and King, 2013), extracellular matrix (ECM) environment (Miyamoto et al., 2004; Luca et al., 2013; Li et al., 2020), as well as mechanical stresses (Davies and Tripathi, 1993). The high correlation coefficients observed for CRC1030 and CRC1414 between *in vitro* and *in vivo* predictions (Figure 5C) are strong indications that the IMITA tumour OoC model is successful in mimicking some of the physiological conditions necessary for the maintenance of the PDX tumour cells. Specifically, the IMITA device enabled 3D culture as tumour spheroids, with perfusion, to maintain low fluid-induced shear stress.

Nonetheless, the IMITA device did not adequately predict the *in vivo* PDX drug responses for CRC935. We postulate this may be due to the lack of ECM support within the IMITA device. Between the 3 patient samples, CRC935 tumour tissues showed a higher amount of ECM (Supplementary Figure S5) compared to CRC1030 and CRC1414. We therefore treated all harvested PDX tumour tissues with collagenase type IV to remove as much ECM as possible to facilitate the uniform packing of tumour cells in the culture chamber array without clogging (Supplementary Figure S4). This approach is similar to that used in previous micro-perfusion chambers incorporating PDX tumour cells (Gheibi et al., 2017). Since the IMITA device utilises micro-pillars to physically pack seeded cells into 3D tumour organoids, they can be prone to uneven distribution of cell seeding and cell clogging which interferes with the packing of the cells and the flow field of medium perfusion. This observation serves as a guide for future iterations of device designs which can easily accommodate a tumour’s native ECM. One potential approach to accommodate ECM-based cell culture, is to leverage the capillary forces generated between hydrogels and the microfluidic channels to create various micro-patterned geometries (Chung et al., 2009; Shin et al., 2012; Menon et al., 2017). Through this approach, the harvested CRCs can be first embedded within the hydrogels before patterning in microfluidic chambers. Being less reliant on micropillars for cell packing, this methodology could avoid the ECM clogging issues within the microfluidic devices, commonly faced in the IMITA device without collagenase treatment. Furthermore, incorporation of fibroblast culture chambers to enable co-cultures within tumour OoCs could increase similarity in predicting drug responses between *in vitro* and *in vivo* models (Kuen et al., 2017; Koh et al., 2019). The demonstrated *in vitro-in vivo* correlative framework could potentially be deployed to assess the suitability of various OoCs in immunotherapy testings by co-culturing of tumour with effector cells such as NK (Subedi et al., 2021) and CAR-T-cells into OoC platforms (Wang et al., 2019).

## Conclusion

One of the key appeals of tumour OoC devices is their potential to reduce the reliance of animal models for cancer drug testing. To achieve this goal, there is a need to systematically validate how well these *in vitro* models predict the drug-response obtained from PDX animal models. In this study, we have successfully established a multiplexed tumour OoC device that can recapitulate a dynamic 3D culture environment to maintain PDX derived primary tumour tissues from 3 CRC patients. This enabled us to systematically compare the drug responses to 5 different SOC chemotherapeutic regimes between the *in vitro* microfluidic tumour OoC model and PDX *in vivo* model. In addition, we established a framework to statistically examine the correlation between ranked drug efficacies based on *in vitro* IC_50_ values and *in vivo* TGI values. It was found that *in vitro-in vivo* correlation coefficient was >0.70 for 2 out of the 3 CRC patients. This indicated the potential application of *in vitro* microfluidic platform in drug screening applications towards personalised cancer treatment.

## Data Availability

The original contributions presented in the study are included in the article/Supplementary Material, further inquiries can be directed to the corresponding authors.
