# Supplementary material for: A comparative study of tumour-on-chip models with patient-derived xenografts for predicting chemotherapy efficacy in colorectal cancer patients
Source: Front Bioeng Biotechnol. 2022 Aug 16;10:952726. doi: 10.3389/fbioe.2022.952726 (PMC9488115; doi:10.3389/fbioe.2022.952726)
Supplement: Supplementary file 1 [file Image1.pdf]

A comparative study of tumour-on-chip models with patient-derived xenografts for predicting chemotherapy efficacy in colorectal cancer patients

Supplementary Information

Louis Jun Ye Ong[1-4$], Shumei Chia[5$], Stephen Qi Rong Wong[5,6], Xiaoqian Zhang[5], Huiwen Chua[5], Jiamin Loo[5], Wei Yong Chua[5], Clarinda Chua[7], Emile Tan[8], Hannes Hentze[9], Iain BeeHuat Tan[5,7,10], Ramanuj DasGupta[5]*, Yi-Chin Toh[1-4]*

1 School of Mechanical, Medical and Process Engineering, Queensland University of Technology (QUT), Level 7, O Block, Gardens Point Campus, Brisbane City QLD 4000, Australia.

2 Centre for Biomedical Technologies, Queensland University of Technology (QUT), Q Block-IHBI, 60 Musk Avenue, Kelvin Grove QLD 4059, Australia.

3 Department of Biomedical Engineering, National University of Singapore, 4, Engineering Drive 3, E4-04-10, Singapore 117583, Singapore.

4 Institute for Health Innovation and Technology, National University of Singapore, 14 Medical Drive, #14-01, Singapore 117599, Singapore.

5 Genome Institute of Singapore, A*STAR, Laboratory of Precision Oncology and Cancer Evolution, 60 Biopolis Street, # 02-01 Genome, Singapore 138672.

6 Biological Resource Centre, Agency for Science, Technology and Research (A*STAR), Singapore

7 National Cancer Centre Singapore, 11 Hospital Crescent, Singapore 169610.

8 Singapore General Hospital, Outram Rd, Singapore 1696089

9 Experimental Drug Development Centre, 10 Biopolis Rd, #05-01/06 Chromos, Singapore 138670

10 Duke-NUS Graduate Medical School, Singapore

[$] Equal contribution

*Corresponding address:

Yi-Chin Toh, Email: yichin.toh@qut.edu.au

Ramanuj DasGupta, Email: dasguptar@gis.a-star.edu.sg

Supplementary Figures

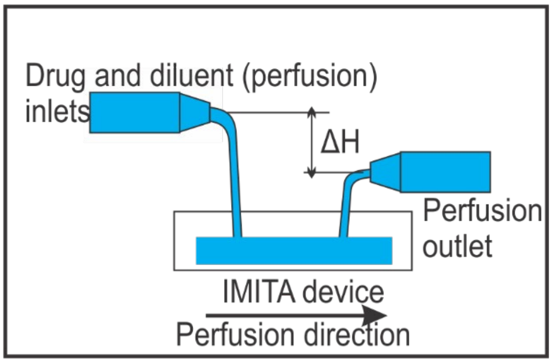

SI Fig 1: Pump-free perfusion setup for IMITA device.

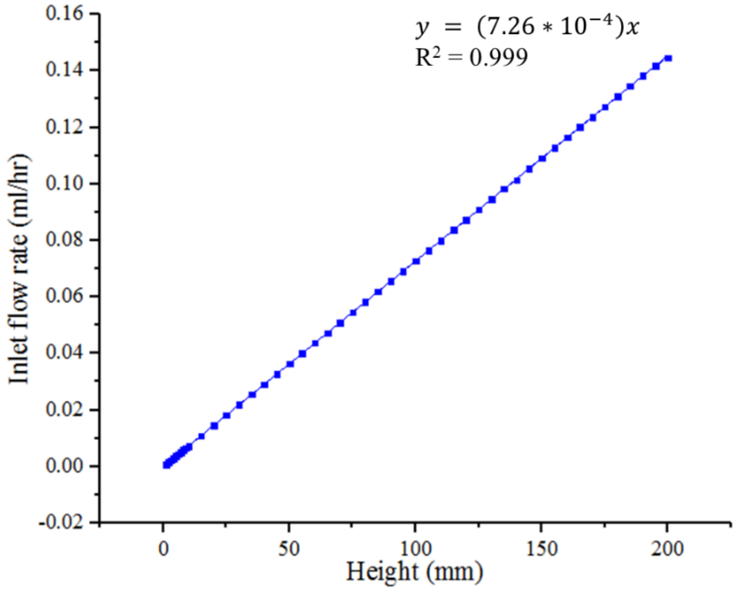

SI Fig 2: CFD simulation of a tunable flow rate by adjusting the height difference between the perfusion inlets and outlet reservoirs.

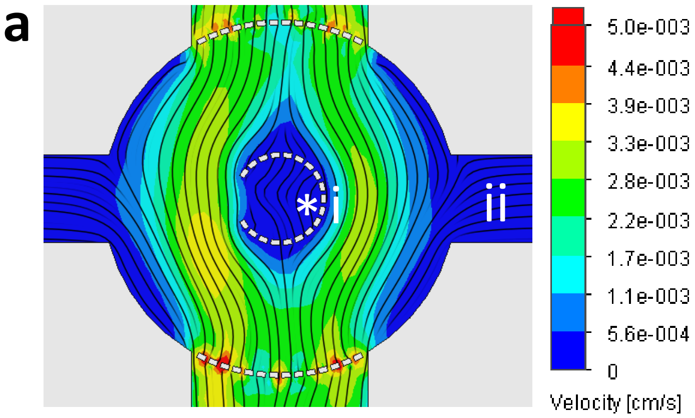

b

Position	Pe		
	5FU	Ox	CPT11
i	32.52	48.78	18.52
ii	2.90	4.35	1.65

SI Fig 3: (a) Flow velocity within one cell chamber of IMITA platform during perfusion culture. CFD simulation is performed at steady state flow conditions with a flow rate of 0.02 mL hr^{-1}. Shear stress at * is estimated to be at 0.04 dynes cm^{-2}. Steady state Peclet number estimation at the center of the culture chamber (i) and the connecting channels (ii). (b) Péclet number (Pe) of different SOC drugs used at key regions of the IMITA device.

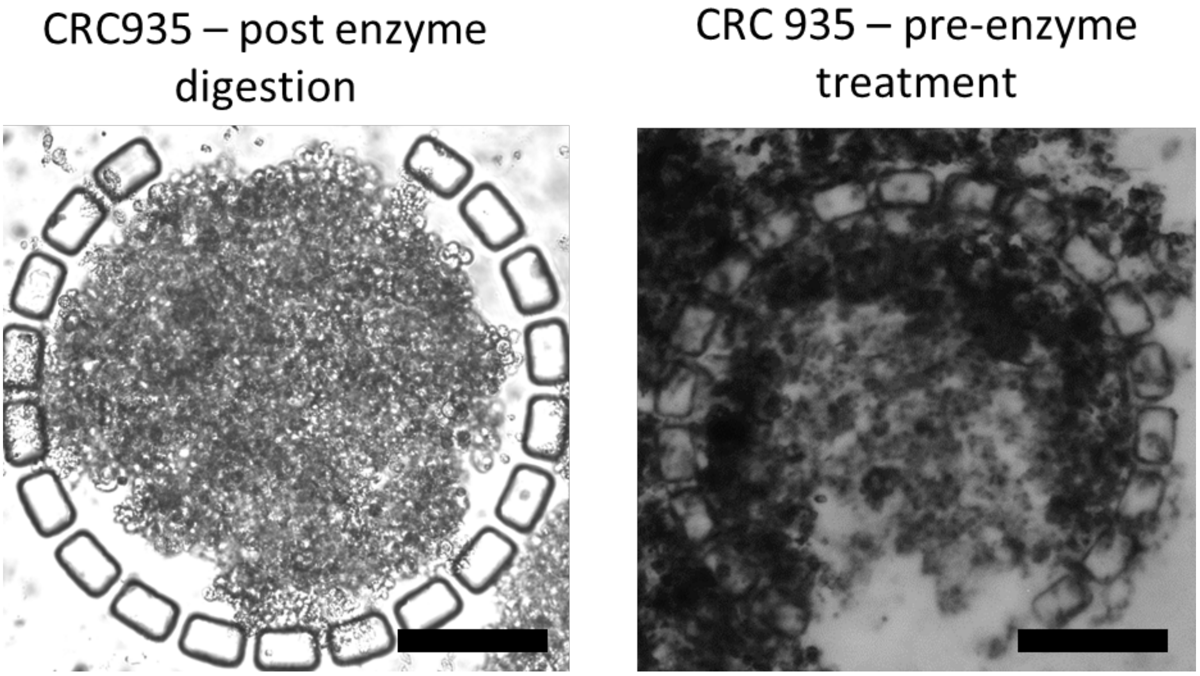

SI Fig 4: Comparison in seeded CRC935 cells after enzymatic digestion and before enzymatic digestion in the IMITA device. Scale = 167 µm.

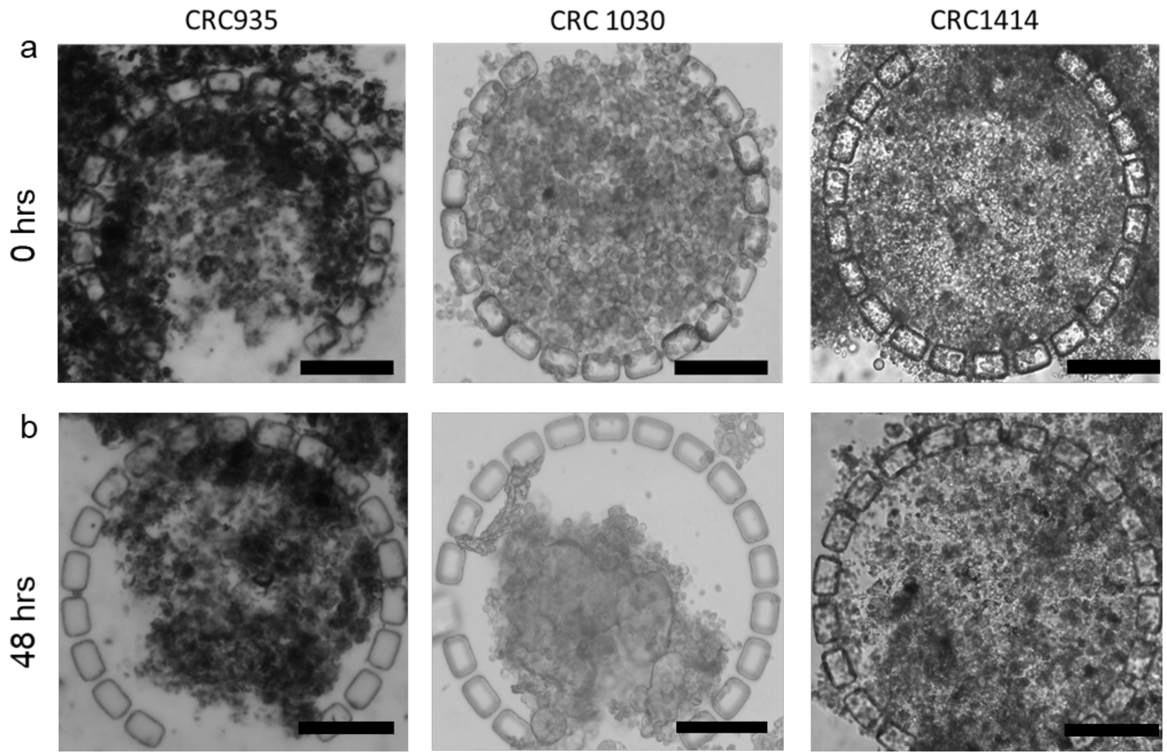

SI Fig 5: Characteristics of the seeded PDX tumour cells in IMITA device. (a) Phase images showing different PDX tumour cells from patients CRC935, CRC1030, and CRC1414 immediately post seeding the IMITA device. (b) Calcein-Am staining of seeded PDX tumour cells from CRC935, CRC1030, and CRC1414 48 hours post seeding. Scale = 167 µm.

a

Mesh scale	Max element size / µm	Min element size / µm	Max element growth rate
Low	16.2041	10.2654	1.2
Med	8.5406	6.4235	1.2
High	4.3657	0.7125	1.2

b

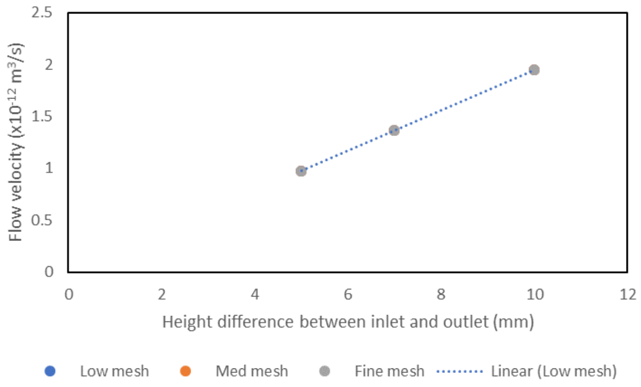

SI Fig 6: Mesh setting of the CFD simulation and mesh independent study. (a) mesh scale setting of the device geometry and (b) mesh independence test for the CFD. A medium-mesh scale is used to reduce computing time for this work.
